# Industrially Contaminated Sites and Health

**DOI:** 10.1155/2014/198574

**Published:** 2014-03-11

**Authors:** Marco Martuzzi, Roberto Pasetto, Piedad Martin-Olmedo

**Affiliations:** ^1^World Health Organization, Regional Office for Europe, European Centre for Environment and Health, Platz der Vereinten Nationen, 1, 53113 Bonn, Germany; ^2^Unit of Environmental Epidemiology, Department of Environment and Primary Prevention, Istituto Superiore di Sanità, Viale Regina Elena, 299, 00161 Rome, Italy; ^3^Escuela Andaluza de Salud Publica, Cuesta del Observatorio 4, 18080 Granada, Spain

According to data collected by the European Environment Agency, Europe has hundreds of thousands of contaminated sites [1], many of them resulting from earlier industrialization and poor environmental management. Past and present activities can cause dispersion and accumulation of countless contaminants, mainly chemicals, to an extent that might affect human health by compromising air quality, altering soil functions, entering the food chain, and polluting groundwater and surface water. Typically, but not always, these stressors occur in localized areas near the point sources and affect local communities.

The assessment of possible health impacts related to sites hosting or having hosted large or intensive production and processing plants (such as chemicals, petrochemicals, manufacturing, waste disposal and treatment, cement, power generation, mining, and metals) is the main focus of this special issue. Such assessments entail considerable challenges, but the scientific literature repeatedly documented these impacts, often substantial. A variety of methods and tools have been developed for the analysis of these studies: the review included in this special issue by M. Pascal et al., for example, describes the available methodologies for assessing the impacts of airborne contaminants around major industrial areas. These and other resources are available and have been applied in numerous investigations, examples of which are also presented in this special issue: L. Pascal et al. report on hospital admissions near a large industrial area in France; O. Breugelmans et al. studied cancer incidence around a steel plant in The Netherlands, using hierarchical Bayesian models; and R. Pirastu et al. applied different epidemiological approaches to study the health profile of residents in the Italian National Priority Contaminated Site in Taranto. All three studies indicate elevated risks for some of the health outcomes considered, in some cases with large excesses.

A number of agents and contaminants examined in these studies are well-established risk factors (e.g., asbestos) and in many cases the evidence provided was instrumental to undertake effective remediation.

Available assessments indicate that industrially contaminated sites represent an important public health issue for several reasons: the large extent of contamination and ensuing health impacts, documented in many contaminated sites; the coexistence of multiple environmental stressors; the concurrence of several residential and/or occupational exposure pathways; the largely unknown interaction with risk factors from the social environment, such as lifestyle (nutrition, tobacco consumption, alcohol, physical activity, and housing quality); and the markedly uneven distribution of the risks that raises issues of health inequality and environmental justice.

However, while the scientific literature on the subject is rich, it is not yet possible to draw a reliable comprehensive picture of the health impacts of contaminated sites, as some questions remain open. Some of these questions are addressed in this special issue.

For a start, the heterogeneity of these sites makes a classification difficult; a common legal definition of contaminated site is not available, nor are common criteria to set up inventories. However, commonalities between certain kinds of areas with respect to the possible health effects exist, and an operational definition of contaminated sites has been proposed as follows: Areas hosting or having hosted human activities which have produced or might produce environmental contamination of soil, surface or groundwater, air, food-chain, resulting or being able to result in human health impacts [2].

The most relevant health outcomes to be considered *a priori* for different types of industrial activities are identified in this special issue, in a paper by R. Pirastu et al. that also presents an approach to describe the health profile of populations living in industrially contaminated sites using routinely collected data.

Epidemiological studies in contaminated sites, especially those focusing on residents (rather than workers), are beset by problems of exposure assessments. Exposure misclassification may in fact compromise the ability of these studies to detect health effects and also, though more rarely, generate false positives. Progress has been made, however, notably through biomonitoring [3] and modelling of dispersion and uptake, as described in this special issue in connection to waste incineration.

To pursue better quantification of the overall health impact of contaminated sites, more systematic data are needed to improve the assessment of human exposure. In this respect, many initiatives at the European level have been developed, especially with soil as an entry point. In this special issue, P. Panagos et al. describe the work undertaken in the framework of the European Union (EU) Thematic Strategy for Soil Protection, where the European Commission has identified soil contamination as a priority for the collection of policy-relevant data at continental scale.

In addition to raising problems with exposure assessment, industrially contaminated sites are often located close to urban areas and/or socially deprived neighbourhoods; this increases the possible extent of the impacts, makes exposure patterns more complex, and is involved in interactions with other health determinants.

Because often the rationale behind these studies is to inform policy decisions, such as remediation, reconversion, or industrial development, there is a need, ideally, to consider all the health stressors occurring in a contaminated site in their entirety, rather than breaking down the question to independent analyses focussing on single contaminants. Such an aspiration to holistic assessments makes the above challenges particularly demanding and should be considered in order to set a research agenda in this domain.

Current efforts include WHO-led expert consultations [2]. Taking stock from important national initiatives and current research in epidemiology, exposure assessment, toxicology, environmental monitoring and modelling, human biomonitoring, risk perception, and other disciplines, consensus has emerged on the urgency of the problem and on possible ways forward and goals for international collaborative work, which include

- producing guidelines on (a) strategies for studying environment and health in contaminated sites and (b) communication strategies;
- developing resources, materials, and training modules on (a) approaches and methods to be applied in different sites and contexts and (b) communication strategies;
- strengthening the methodology on exposure assessment, in particular, on biomonitoring and through the food-chain;
- implementing health assessments that include detailed analyses of population subgroups, in particular, children;
- planning a system to collect data and produce comparative analyses of the health impact of different sources of contamination within and among different countries, allowing for the inclusion of socioeconomic factors.

In conclusion, on the basis of the accumulating evidence on the relevant health impacts (including the contributions contained in this special issue), the need for consistent policies on remediation, and the compelling dimension of the underlying health inequalities, the issue of contaminated sites and health can be considered of priority in the environment and health domain. Existing resources should be further developed and deployed to tackle this important question.

*Marco  Martuzzi* *Marco  Martuzzi* *Roberto  Pasetto* *Roberto  Pasetto* *Piedad  Martin-Olmedo* *Piedad  Martin-Olmedo*
